# Bibliometric Analysis of 30 Years of Scientific Publications Related to Low-Flow Anesthesia

**DOI:** 10.3390/healthcare14081020

**Published:** 2026-04-13

**Authors:** İsmet Çopur, Hüseyin Özçınar, Turan Evran

**Affiliations:** 1 Department of Anesthesiology and Reanimation, Faculty of Medicine, Pamukkale University, 20700 Denizli, Turkey; icopur@pau.edu.tr; 2 Department of Computer Education and Instructional Technology, Faculty of Education, Pamukkale University, 20700 Denizli, Turkey; huseyinozcinar@gmail.com

**Keywords:** bibliometric analysis, closed system anesthesia, environmental impact in anesthesia, low-flow anesthesia, sustainable anesthesia practices

## Abstract

**Background:** This study aims to conduct an analysis of the literature on low-flow anesthesia published over the past 30 years, identifying the most productive countries, institutions, authors, and journals; uncovering the intellectual structure of the field through the most influential publications, authors, and journals; and visualizing thematic clusters and evolving research trends based on keyword analyses. **Methods:** This retrospective bibliometric study analyzed scientific publications on low-flow anesthesia indexed in the Science Citation Index Expanded (SCIE) of the Web of Science Core Collection (WoSCC) between 1993 and 2024. Articles were classified by countries, institutions, journals, and researchers, and the number of studies and citations were determined. Co-citation analysis and keyword co-occurrence analysis were performed to map thematic clusters and intellectual structures. **Results:** A total of 260 articles met the inclusion criteria. The United States led with 39 publications, followed by Turkey (33) and Japan (27). The most productive institution was Northwestern University (USA), and the most published journal was *Anesthesia & Analgesia*. The most prolific authors were André M. De Wolf and Jan F. A. Hendrickx, while co-citation analysis identified Edmund I. Eger II and Hiromichi Bito as the most influential authors based on centrality metrics. MDS and trend topic analyses revealed prominent keywords including “closed loop”, “remifentanil”, “sevoflurane”, “bispectral index”, “EEG analysis”, “pharmacokinetics”, “absorbent”, “performance”, and “FGF” (fresh gas flow). **Conclusions:** The United States leads the field of low-flow anesthesia in both publication count and citations. Trending terms such as “closed loop,” “performance,” “remifentanil,” “sevoflurane,” “bispectral index,” “EEG analysis,” “FGF,” and “absorbent” reflect the current research directions in this field.

## 1. Introduction

Low-flow anesthesia has been used since the early 1950s as a technique in which the flow rate of fresh gas is 1 L/min or less [1,2,3]. Low-flow anesthesia is a method applied for more efficient use of anesthetic gases and reducing oxygen consumption. In this method, concepts such as “rebreathing system” and “closed-circuit anesthesia” refer to systems that allow the recovery of gases inhaled by the patient and have been applied in anesthesia practice since the mid-20th century. These systems aim to minimize consumption by putting the used anesthetic gases back into circulation as much as possible. In the 1990s, it was also called “gas-saving anesthesia” [4,5]. The low-flow anesthesia’s advantages include cost-effectiveness, reduction in anesthetic gas consumption, the minimization of gas emissions released to the environment, and the preservation of heat and humidity of inspired gases [2,4]. Since this technique may have some risks such as hypercapnia and hypoxia, it should be applied carefully in anesthesia practice and regular monitoring should be performed [2,4]. First, it started to be applied with the old-generation volatile anesthetics such as halothane and enflurane, then with the development of safer and more effective agents such as isoflurane, sevoflurane and desflurane, low-flow anesthesia has gained an important place in modern anesthesia practice [6]. Techniques such as minimal flow, closed-circuit systems and respiration systems increase patient safety and reduce environmental impacts by ensuring the effective use of anesthetic gases during low fresh gas flow. Global concern over the environmental impact of volatile anesthetic agents, especially their contribution to greenhouse gas emissions, has prompted international health systems to prioritize sustainable anesthesia practices, including the adoption of low-flow techniques. In recent years, the positive effects of low-flow anesthesia techniques on environmental sustainability have become one of the main reasons why this method is preferred, and various studies have been conducted on this topic [7].

Since the mid-20th century, bibliometric analyses have been extensively utilized to investigate the production dynamics and developmental trajectories of the scientific literature across medical and health sciences [8]. Bibliometric analyses provide important insights into research trends, knowledge gaps, and future directions within a scientific field. Bibliometrics aims to evaluate the impact of these publications in the scientific world by conducting a numerical analysis of scientific publications. In this evaluation, two basic methods are used: performance analysis and science mapping. Performance analysis measures the publication performance of scientists, institutions and countries, while science mapping examines the relationships between publications in order to reveal the intellectual and social structures of a research field. In bibliometric analyses, examining country, author, and journal data not only quantifies scientific productivity but also reveals the geographic regions, research groups, and publication platforms where this productivity is concentrated. By uncovering the interactions among these, it helps determine the direction of knowledge flow, which allows researchers to identify knowledge gaps in the literature and predict future research trends [9,10]. Bibliometric studies are increasingly being used to identify research gaps and provide strategic direction for future research [11]. In recent years, bibliometric analyses conducted in rapidly developing medical fields such as anesthesiology make it possible to comprehensively evaluate the knowledge in this field and identify important information deficiencies [12]. However, a review of the current literature on low-flow anesthesia reveals a significant gap in studies that systematically map the evolution, impact, and collaborative landscape of research in this field, particularly those analyzing contributing institutions, leading researchers, and patterns of scientific collaboration with a specific focus on low-flow anesthesia. The aim of this study is to conduct a bibliometric analysis of the literature on low-flow anesthesia published over the past 30 years, with the objectives of identifying the most productive countries, institutions, authors, and journals; uncovering the intellectual structure of the field through the most influential publications, authors, and journals; and visualizing thematic clusters and evolving research trends based on keyword analyses.

## 2. Materials and Methods

### 2.1. Working Design

This study was designed as a retrospective bibliometric analysis, examining scientific publications related to low-flow anesthesia in the field of anesthesiology between 1993 and 2024. Consistent with previous similar studies, data were obtained from the SCIE of WoSCC, which was selected as the data source to enhance the reproducibility of the study [12,13]. Ethics committee approval was not required for this study because only publicly available data were used.

Although alternative databases such as Scopus (Elsevier), PubMed/MEDLINE (NLM), Dimensions (Digital Science) and Google Scholar offer broader journal coverage, the SCIE of WoSCC was preferred because of its selective indexing structure, its focus on high-impact peer-reviewed journals, the standardized quality of its bibliographic metadata, and its widespread use as a data source in bibliometric analyses of the biomedical and anesthesiology literature [12,13,14].

As previously reported, the substantial absence of abstracts, keywords and indexing information in publications included in the SCIE of WoSCC before 1990, together with the marked improvement in data availability after 1990, has been identified as an important methodological limitation that can influence the results of bibliometric analyses. In addition, further sources of bias such as author name ambiguity and changes in database coverage over time reinforce this concern [15]. Furthermore, with the introduction of modern volatile anesthetics into clinical practice from the 1990s onward, the literature was considered to have become more structured, accessible and amenable to systematic analysis; accordingly, the study period was set to cover the years 1993–2024.

### 2.2. Inclusion Criteria

Only English-language original articles and review articles published between 1993 and 2024 in journals indexed in the SCIE of WoSCC were included in the analysis.

### 2.3. Exclusion Criteria

The identified studies were examined manually; studies that dealt with non-human subjects (for example, veterinary research) or whose title and subject content were incompatible were excluded.

#### 2.3.1. Data Collection and Processing

The search strategy was implemented in the SCIE of WoSCC using the advanced Boolean interface. Two OR-expanded term sets were first created:

Set 1 (Flow terminology) = TI OR AK contains any of “reduced flow”, “low flow”, “minimal flow”, “rebreathing system”, “closed system”.

Set 2 (Anesthesia and agents) = TI OR AK contains any of “anesthesia”, “anesthetics”, “sevoflurane”, “desflurane”, “isoflurane”.

The final query was Set 1 AND Set 2, restricted to articles and review articles indexed in the SCIE of WoSCC. All Boolean terms were agreed upon by a panel of seven consultant anesthesiologists to maximize terminological coverage of low-flow anesthesia (Table 1).

To safeguard against data loss and any future index revisions, we exported the full record and cited-reference data for every document retrieved by our SCIE of WoSCC query on 15 September 2024. For each article, detailed information such as publication year, title, country, institution, journal, DOI, author name and author keywords taken from the SCIE of WoSCC were transferred to Microsoft Excel (v16.0, Microsoft Corporation, Redmond, WA, USA) and saved. To ensure analytical consistency, spelling variations in key metadata fields—journal titles, country names, institutional affiliations, author names, and keywords—were harmonized in Microsoft Excel, and the cleaned dataset was then exported in BibTeX format. Data extraction operations were performed by two independent researchers in order to increase reliability and consensus was reached on the data. In contradictory cases, the final decision was made by taking the opinion of a third researcher.

#### 2.3.2. Data Analysis and Statistical Methods

In this study, co-citation analysis, one of the bibliometric analysis methods, was used to examine the impact of leading publications, authors, and journals related to low-flow anesthesia. These analyses, conducted at the document, author, and journal levels, revealed the intellectual map of the field by uncovering the relationships among its most influential contributors.

All network- and dimensional-reduction analyses were carried out in R (v4.2.2, R Foundation for Statistical Computing, Vienna, Austria) within RStudio (v2022.12.0-353, Posit Software, PBC, Boston, MA, USA) using the open-source package bibliometrix v4.0.1 and its graphical front-end biblioshiny [10]. For the co-citation network, we retained the 50 most globally cited documents (Number of Nodes = 50), discarded singletons (Remove Isolated Nodes = Yes), applied association normalization, required at least two shared references per edge (Minimum Number of Edges = 2) and set the repulsion force to 0.1. Community detection used the Leiden algorithm (resolution = 1.0; CPM modularity; 200 random starts; iterative until convergence) as implemented in bibliometrix. For the keyword co-occurrence network (field = Keyword Plus, author keywords or abstract words), we again capped the graph at 50 nodes, kept the same edge and repulsion thresholds, and identified clusters with Walktrap (six-step random walk). Conceptual structure analyses employed MDS on the cosine-similarity matrix of Keyword Plus, author keywords or abstract, restricting the map to the top 50 terms and cutting the dendrogram at k = 3 clusters. The trend topics and thematic-evolution panels followed bibliometrix defaults except where noted: Number of Words = 250, Minimum Cluster Frequency = 5 per 1000 documents, Weight Index = “Inclusion index weighted by word occurrences”, Min Weight Index = 0.1, Label Size = 0.3, Maximum Labels per Cluster = 3.

In this study, co-citation analysis is applied separately for document, author, and journal networks, with nodes representing documents, authors, and journals, respectively. To explore the roles and influence of these entities within their networks, we employ widely used metrics such as PageRank, betweenness centrality, and closeness centrality. These measures are fundamental in bibliometric research and play a crucial role in co-citation network analysis [16].

PageRank: This criterion is calculated according to the number of links received by a node (article, author or journal) and the importance of these links. A high PageRank indicates that the node is referenced frequently and has established strong relationships with other nodes. Moreover, PageRank does not only consider how many citations a publication receives, but also evaluates the source of those citations. This allows the identification of highly influential works that shape the overall direction of the field, beyond simple citation counts.

Betweenness: This criterion refers to the degree to which an author, keyword or article can act as a bridge between different groups on the network. A high betweenness value indicates that this node plays a key role in the flow of information and provides connections between other groups. Thus, betweenness was used not only to assess influence, but also to help identify studies that contribute to the integration of knowledge across interdisciplinary domains and promote innovation and collaboration within the research field.

Closeness: This criterion shows the average distance of a node to other nodes in the network. A high closeness value indicates that the node is located centrally within the network and provides quick access to other nodes. Therefore, closeness was used to identify studies or authors that are positioned at the heart of the scientific network and play a key role in efficiently connecting diverse sub-topics, accelerating the spread of knowledge across the field.

The co-citation networks created to determine the subdomains of the field were analyzed by the Leiden clustering method. Consistent with the default settings of bibliometrix executed in RStudio under R, the co-citation network was constructed from the 50 documents with the highest global citation counts, and communities were detected using the Leiden algorithm (resolution = 1.0). Leiden clustering analysis has been preferred as an effective algorithm that can detect communities or subgroups in large and complex data sets in detail, providing high quality results [17]. This algorithm groups articles, authors and journals according to similar topics and assigns a cluster number to each group. There are stronger links between articles, authors and journals in the same cluster, indicating that these items focus on similar themes or topics.

#### 2.3.3. Keyword Co-Occurrence Analyses

In this study, the Multidimensional Scaling (MDS) method was applied in order to examine how the concepts and terms used in the low-flow anesthesia literature are grouped and to understand the thematic structure of the field. MDS analysis visualizes the similarities or differences among elements in complex datasets within a two- or three-dimensional space, clustering terms with high similarity and representing the strength of their relationships as distance. With this method, the words with high correlation were collected under certain clusters and the main themes within the field were revealed. Author keywords, Keyword Plus terms and article abstracts were analyzed separately and the clusters formed were visualized in color. These clusters obtained by the MDS method clearly reveal the contexts in which the terms in the articles are used together and the thematic trends. In addition, trend topic analysis, one of the bibliometric analysis methods, has been applied and visualized in order to identify the outstanding issues in the low-flow anesthesia literature over time. In this analysis, in addition to author keywords, Keyword Plus and article abstract terms were used to determine the broad research themes and changing trends in the literature. Terms were standardized, singular–plural differences were resolved, and only keywords used at least five times were included in the analysis.

## 3. Results

**Figure 1:**
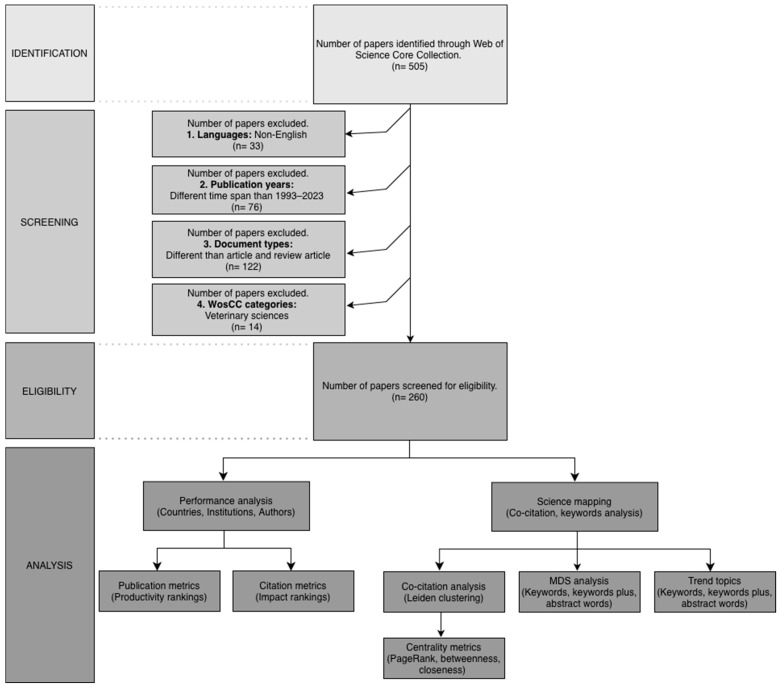
PRISMA-style flow diagram for study selection and bibliometric analysis process.

In this study, a bibliometric analysis of the published studies on low-flow anesthesia in the last 30 years was performed. In the first search conducted at the SCIE of WoSCC, 505 publications were identified. However, 33 publications that are not in English, 76 publications that were excluded from the analysis period and 14 publications belonging to the veterinary field were excluded from the study. Only 260 publications of the “article” and “review article” types were included in the analysis (Figure 1).

According to the citation analysis conducted for publications in the field of low-flow anesthesia, the most influential countries, institutions and authors have been identified (Table 2). In the last 30 years, the most articles have been produced by the USA (39 articles, 15%). Turkey ranks second with 33 articles (12.7%) and Japan ranks third with 27 articles (10.4%). Belgium and Germany also make significant contributions with 16 articles each (6.2%). At the institutional level, Northwestern University (USA) is the institution with the most publications with 14 articles. The University of Ghent (Belgium) is in second place with 13 articles, and the University of Washington (USA) is in third place with 12 articles. The most widely published journal is *Anesthesia & Analgesia* with 24 articles, followed by *Anesthesiology* (23 articles) and *Acta Anaesthesiologica Scandinavica* (19 articles).

**Figure 2:**
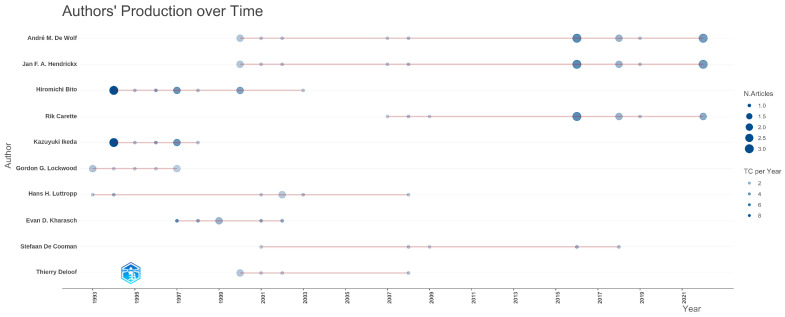
Authors’ publication productivity by year.

The most prolific authors are De Wolf and Hendrickx with 15 articles each; Bito (11 articles), Carette (11 articles) and Ikeda (8 articles) are also among the authors who have made significant contributions to the field. There is a significant increase in the publication productivity of authors between 1995–2000 and 2015–2020 (Figure 2).

**Figure 3:**
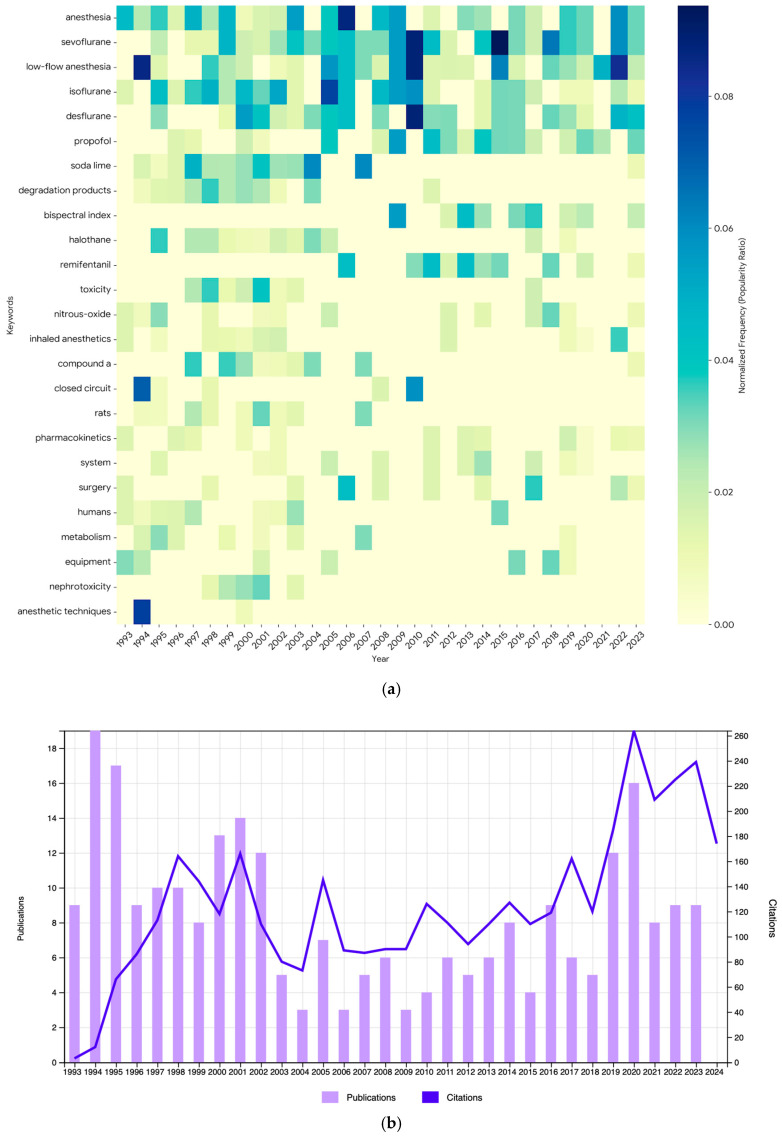
(**a**) Heatmap of keyword frequency by year. (**b**) Number of publications and citations by year.

A heatmap of keyword frequency by year indicates that terms such as “low-flow anesthesia,” “sevoflurane,” and “bispectral index” have shown increased usage in recent years (Figure 3a). The USA, Japan and Germany stand out in the average number of citations; USA ranks first with 929 citations (14.43%), Japan ranks second with 703 citations (10.91%) and Germany ranks third with 338 citations (5.25%). Significant increases were observed in the number of citations, especially in the years 1997–1998, 2020 and 2022 (Figure 3b).

The highest citations among the institutions were determined as Northwestern University (14 citations, 0.22%), Ghent University (13 citations, 0.20%) and the University of Washington (12 citations, 0.19%). In terms of journals, *Anesthesiology* is the journal that receives the most citations with 912 citations; *Anesthesia & Analgesia* is in the second place with 779 citations and the *British Journal of Anaesthesia* is in the third place with 321 citations. Other important journals include *Acta Anaesthesiologica Scandinavica* (274 citations), *Anaesthesia* (232 citations) and *Anaesthesia & Intensive Care* (154 citations).

The authors with the highest number of citations are Hiromichi Bito (99 citations, 1.54%), Kazuyuki Ikeda (87 citations, 1.35%) and Evan D. Kharasch (51 citations, 0.79%), who stand out as the authors with the highest number of citations. In particular, Hiromichi Bito’s study titled “Closed-Circuit Anesthesia With Sevoflurane in Humans” and Evan D. Kharasch’s article titled “Assessment of Low-Flow Sevoflurane and Isoflurane Effects on Renal Function” published in the journal *Anesthesiology* in 1997 are among the most cited studies (Table 3).

When the network centrality metrics and citation effect of low-flow anesthesia publications are examined, Alan D. Baxter (1997) stands out in both PageRank (0.027) and betweenness (260.571) values in the first cluster, while Murat Bilgi (2011) has the highest closeness (0.003) value. In the second cluster, Michel M. R. F. Struys (2001) has the highest PageRank value (0.024), while Thomas M. Hemmerling (2010) attracts attention with the highest betweenness (0.183) and Ngai Liu (2011) with the highest closeness (0.002) values. In the third cluster, Hiromichi Bito (1994) has the highest PageRank (0.033) and Eric J. Frink (1992) has the highest betweenness values (354.139), while Bito (1994), Michio Morio (1992), Christopher T. Gonsowski (1994) and Bito (1995) share the highest closeness values (0.003) (Table 4).

**Figure 4:**
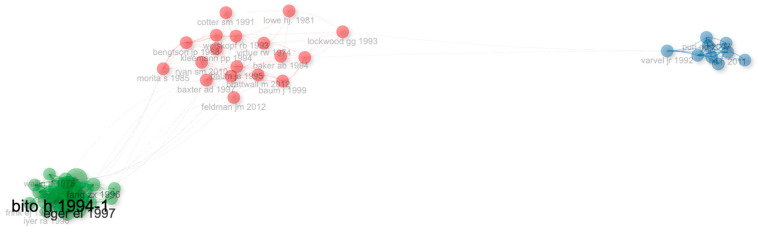
Network visualization of influential publications.

As a result of the clustering analysis performed with the Leiden algorithm, three main clusters were determined in the literature of low-flow anesthesia. The green cluster, which includes studies such as Bito (1994) and Edmund I. Eger II (1997), covers studies that are in a central position in the literature. The red and blue clusters contain publications by authors such as James R. Varvel (1992) and Kharasch (1997) (Figure 4).

**Figure 5:**
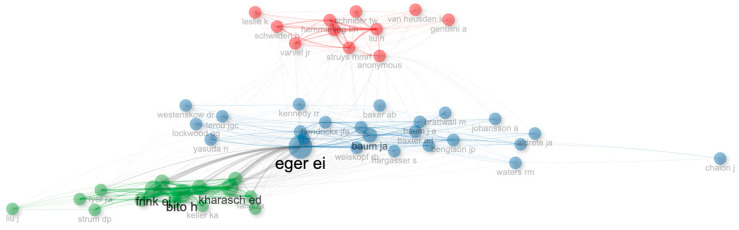
Network visualization of influential authors.

When the centrality criteria of the authors in low-flow anesthesia research were examined, the highest PageRank value in the first set belonged to Liu (0.031), the highest betweenness value belonged to Struys (109.803), and the highest closeness value belonged to Hemmerling (0.003). In the second set, Eger stands out in both PageRank (0.046) and betweenness (580.967) values, while the authors with the highest closeness value are Jan A. Baum, William W. Mapleson, Jan Baum and Jan F. A. Hendrickx (0.008). In the third set, Bito has the highest PageRank (0.039) and Frink has the highest betweenness (103.913) values, while the author with the highest closeness value is again Bito (0.007) (Table 5). These centrality measures reflect different aspects of the authors’ influence within the field. High PageRank values indicate that their work has shaped the direction of the field; high betweenness values suggest that the author serves as a bridge between different research domains; and low closeness values imply that the author occupies a central position within the literature. When evaluated collectively, these metrics demonstrate that authors such as Eger and Bito have played a pivotal role in the development of low-flow anesthesia research. According to the Leiden clustering analysis, the most effective studies in the low-flow anesthesia literature are divided into three main clusters. In the green cluster, Eger and Bito stand out as authors who contribute to knowledge accumulation and establish strong research connections. In the blue cluster, Baum and Lockwood play a central role with a wide network of collaborations, while in the red cluster, Liu and Schmidt stand out with their strong collaborations among themselves, with less sharing centrally (Figure 5). In the clustering analysis, the blue cluster represents research areas focused on pharmacological properties, the red cluster on the effects on organ systems, and the green cluster on monitoring and control systems.

**Figure 6:**
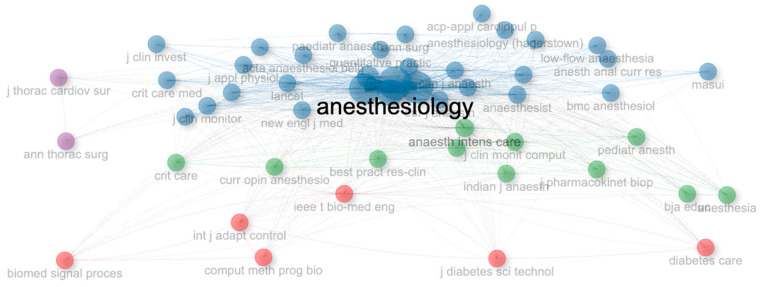
Network visualization analysis of influential journals.

When the centrality measures of journals in low-flow anesthesia research are examined, in the second cluster, the journal *Anesthesiology* has the highest PageRank (0.099) and betweenness (82.163) values; the journals with the highest closeness value include *Anesthesiology*, *Anesthesia & Analgesia*, *British Journal of Anaesthesia*, *Anaesthesia*, *Acta Anaesthesiologica Scandinavica*, *Journal of Clinical Anesthesia*, *European Journal of Anaesthesiology* and *Canadian Journal of Anaesthesia* (0.013). In the third set, *Anaesthesia & Intensive Care* had the highest betweenness (488.373) and the *Canadian Journal of Anesthesia* had the highest closeness (0.013), while the *Journal of Clinical Monitoring and Computing* had the highest PageRank (0.021) (Table 6). PageRank, betweenness, and closeness centrality measures quantitatively reveal the influence and position of each journal within the low-flow anesthesia literature. The high PageRank and betweenness values of the journal *Anesthesiology* indicate that it occupies a central role in the field and serves as a bridge between different research topics. The high betweenness value of *Anesthesia & Intensive Care* suggests that this journal connects various areas of expertise. The journal *Anesthesiology* stands out as the source that makes the most contribution to the low-flow anesthesia literature by being located in the central position of the network. In the figure, the journals in the blue clusters (*Anesthesiology*, *British Journal of Anaesthesia*, *Anesthesia & Analgesia*) have the highest centrality scores in the literature, representing the sources that receive the most citations and interact the most. Journals such as *Biomedical Signal Processing* and *Journal of Thoracic and Cardiovascular Surgery*, which are located in red and purple clusters, show publications related to different areas of expertise in low-flow anesthesia research (Figure 6).

Thematic clusters identified through MDS analysis were categorized into three color-coded groups (blue, red, green) based on semantic similarity and co-occurrence patterns of terms across author keywords, Keyword Plus, and abstract words (Table 7).

**Figure 7:**
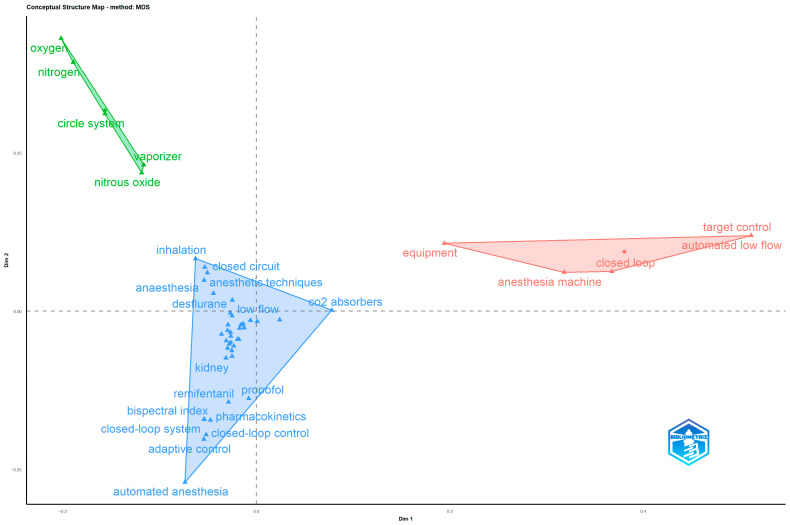
MDS analysis of author keywords.

According to the MDS analysis performed with author keywords, it was found that terms related to automatic and closed-circuit systems such as “automated anesthesia”, “adaptive control” and “closed-loop control” were included in the blue cluster, as well as terms related to pharmacological agents used in low-flow anesthesia and drug management such as “remifentanil,” “propofol” and “desflurane”. This indicates that automated anesthesia management and pharmacological agent optimization have emerged as significant sub-disciplines within low-flow anesthesia research. In the red cluster, it was observed that there are terms related to the equipment and devices used in the field of anesthesia, such as “anesthesia machine” and “equipment”. An analysis of the relationships between the terms in this cluster and those in other clusters revealed that the terms “anesthesia machine” and “equipment” exhibited high betweenness values. This suggests that equipment technology serves as a bridge between pharmacological applications and respiratory system designs, highlighting the importance of interdisciplinary research. On the other hand, it was found that the terms belonging to respiratory and circuit systems such as “circle system” and “vaporizer” are included in the green cluster, unlike the first two clusters (Figure 7).

**Figure 8:**
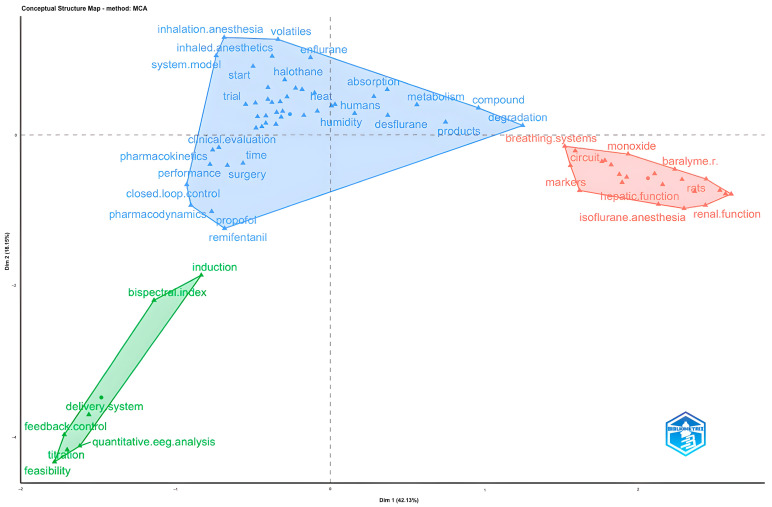
MDS analysis of Keyword Plus words.

According to the MDS analysis of Keyword Plus terms, it was found that the blue cluster contains terms related to anesthetic agents and pharmacological properties such as “inhalation anesthesia,” “halothane,” “desflurane” and “propofol,” as well as terms that can be evaluated in anesthesia management and control systems such as “closed-loop control” and “performance”. In addition, it was determined that terms related to pharmacokinetics such as “compound,” “degradation,” “absorption” and “metabolism” are also included in this cluster. In the red cluster, it was observed that there are terms such as “hepatic function” and “renal function” that express the effects on organ systems. In the green cluster, there are terms related to monitoring and monitoring systems such as “bispectral index” and “quantitative EEG analysis”, as well as terms related to control and application methods such as “feedback control” and “titration”. In addition, it was found that terms that can be evaluated in clinical practice subjects such as “feasibility” and “induction” are also found in this cluster (Figure 8).

**Figure 9:**
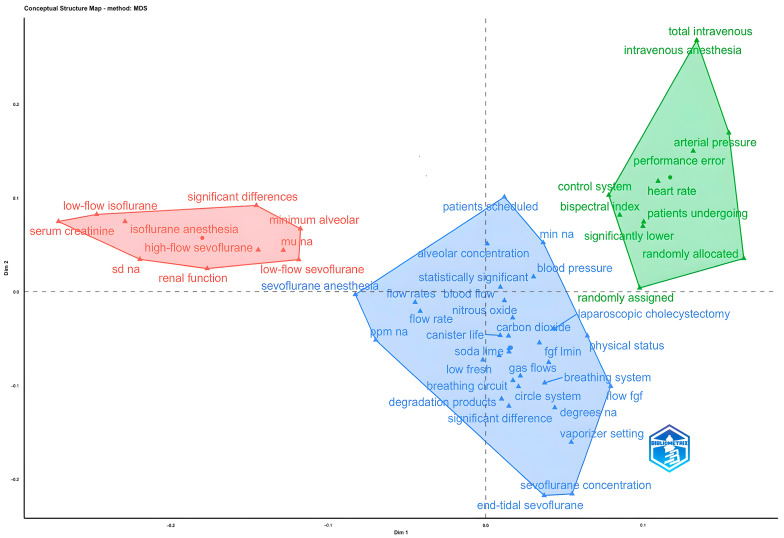
MDS analysis of abstract words.

According to the MDS analysis of abstract terms, it was determined that terms related to circuit systems such as “flow rates” and “breathing circuit”, application and monitoring of anesthetic gases such as “sevoflurane concentration” and “end-tidal sevoflurane” are concentrated in the blue cluster. In addition, it was observed that terms related to closed-circuit anesthesia and gas exchange, such as “carbon dioxide” and “nitrous oxide”, are also included in this cluster. In the red cluster, it was found that there are terms related to anesthesia agents such as “low-flow isoflurane” and “sevoflurane anesthesia” and methods of administration, effects on organ systems such as “renal function” and “serum creatine”. This set also includes terms related to statistical indicators such as “significant differences”. In the green cluster, it was determined that anesthesia methods such as “total intravenous anesthesia” and “randomly allocated”, as well as scientific research methods and terms related to organ systems such as “arterial pressure” and “heart rate”, are concentrated. In addition, it was found that terms related to control and performance criteria such as “control system” and “bispectral index” were also observed in this cluster (Figure 9).

**Figure 10:**
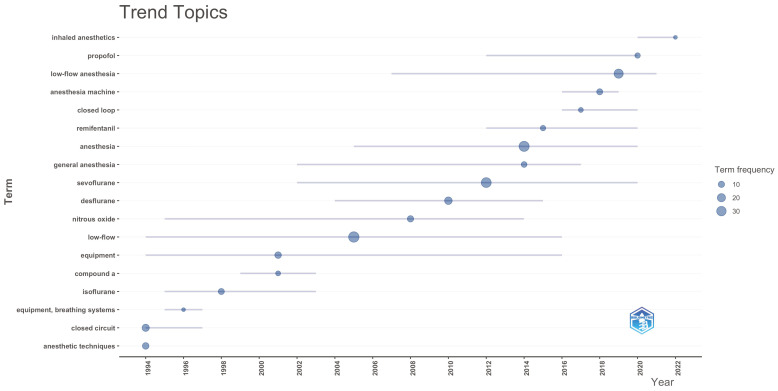
Trend analysis of author keywords.

**Figure 11:**
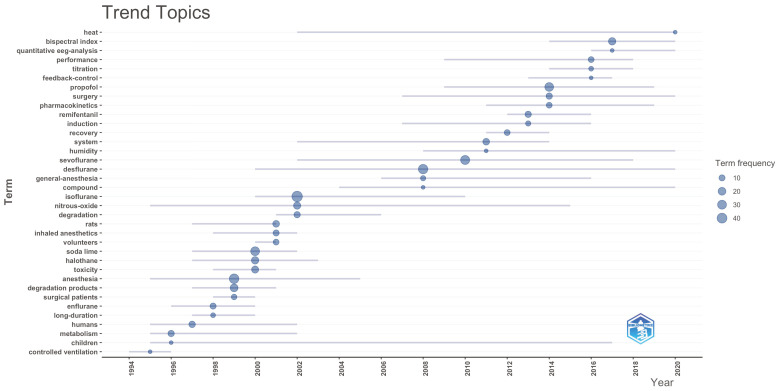
Trend analysis of Keyword Plus words.

**Figure 12:**
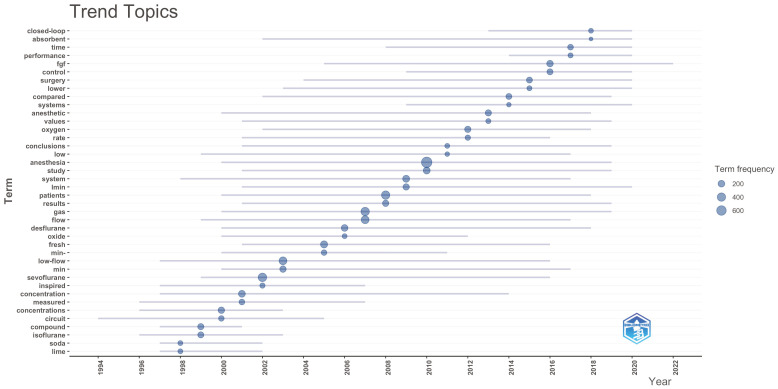
Trend analysis of abstract words.

In the trend topic graphs, it was observed that the terms “propofol”, “closed loop”, “remifentanil” and “sevoflurane” were frequently used trend words in the last 5 years among the author keywords (Figure 10). When we look at the Keyword Plus words, “heat,” “bispectral index,” “EEG analysis” and “pharmacokinetics” come to the fore (Figure 11). Among the abstract words, it was determined that words such as “closed-loop,” “absorbent,” “performance,” “time” and “FGF” have been trending in the context of low-flow anesthesia (Figure 12). These findings indicate that, in recent years, topics such as pharmacological agents, automated control systems, and neuro-monitoring have come to the forefront in low-flow anesthesia research, and that research trends in the field have increasingly concentrated around these themes.

## 4. Discussion

This study, in which we aim to map the information structure of the scientific literature on low-flow anesthesia, to reveal the subfields and their relationships, and to provide predictions about future research directions, is the first study to conduct a bibliometric analysis of this field as far as we know. Our findings show that the United States is the leader in scientific publication productivity in this field with three institutions and two journals, and holds an important position in the number of citations, with four institutions and three journals. Canada and Sweden also make remarkable contributions in terms of the number of publications; Canada is among the other leading countries contributing to this field with two institutions, and Sweden with one institution and one journal. Belgium, the United Kingdom and Germany are the leading countries in Europe in terms of citation numbers; Belgium makes significant contributions to the literature with two institutions and the United Kingdom with two journals. This geographical distribution suggests the potential for broader international collaboration to increase research diversity and impact.

Research by Bould and colleagues reported that, in highly cited anesthesia journals, the United States is ahead in the production of original publications, but the number of original articles decreases as the income level decreases [18]. Similarly, Doğan and Karaca’s study also identified the United States as the most important country and indicated the journals *Anesthesiology* and *Anesthesia & Analgesia* as the main sources [12]. Our findings are generally consistent with this literature. Although Turkey is not among the high-income countries, it stands out in the number of publications after the United States; however, it cannot show the same success in the citation ranking. This situation suggests that Turkey’s scientific impact is relatively limited. On the other hand, Japan, a high-income country, has not been able to enter the top 10 in the rankings of the most influential journals and institutions, despite the contributions of prolific authors Bito and Ikeda. It is thought that this condition is caused by the fact that leading Japanese authors turned to journals that are dominant in the anesthesia literature. Additionally, factors such as national publishing preferences, language barriers, and limited inclusion in internationally indexed databases may also contribute to this underrepresentation.

In Frink’s study, in which he made a clinical comparison of sevoflurane and isoflurane in healthy individuals, it is emphasized that volatile anesthetics interact with CO_2_ absorbents, especially soda lime, under conditions of low-flow anesthesia [19]. This study has the highest betweenness value in the field of low-flow anesthesia and serves as an important bridge by establishing strong links with other studies in the field. In addition, it has been published in *Anesthesia & Analgesia*, one of the prestigious journals that stand out in terms of centrality criteria. Another study by Bito shows that breakdown products such as compound A and compound B are formed by interacting with soda lime in cases where low-flow sevoflurane anesthesia is applied for more than 5 h, and examines the effects of these products on renal and hepatic functions [20]. This study has the highest PageRank value in the literature of low-flow anesthesia and is of central importance in the literature. From the late 1990s to the early 2000s, Bito made significant contributions to the literature through studies published in prestigious journals such as *Anesthesiology*, *Anesthesia & Analgesia*, *British Journal of Anaesthesia* and *Journal of Clinical Anesthesia* on the safety and effectiveness of low-flow anesthesia. Alan D. Baxter’s study “Low and Minimal Flow Inhalational Anesthesia” highlights the advantages of these techniques such as cost reduction, reducing environmental impact, and humidification of gas and patient safety [21]. This study, published in the *Canadian Journal of Anesthesia*, is a bridge in the literature with a high betweenness value. Michel Struys’ study of the target-controlled administration of anesthetics appears to be at the center of research related to low-flow anesthesia and emphasizes the clinical benefits of controlled anesthesia recovery with rapid induction and stable maintenance [22]. Edmund I. Eger II’s studies stand out with their high betweenness, PageRank and closeness values, and are notable for their experimental research on the safety of low-flow sevoflurane anesthesia and the toxicity of the compound A in patients with chronic renal failure [23,24,25]. Current bibliometric analyses conducted by Doğan and Karaca [12] and Chen et al. [26] show that the journals *Anesthesiology*, *Anesthesia & Analgesia*, *British Journal of Anaesthesia* and *Anaesthesia* are among the most cited sources in the field of anesthesiology. Our study supports these findings and reveals that these journals occupy a central position in all the anesthesia literature [12,26]. It is believed that the data obtained by co-citation analysis are effective in determining the centrality of the field and may also be useful in similar studies.

As a result of clustering author keywords, Keyword Plus terms and abstract terms with the MDS analysis method, word groups collected under certain contexts in the low-flow anesthesia literature have been formed. As Boubaker also noted, the MDS method is an effective tool for determining sub-topics based on keywords [27]. In this context, our analysis has revealed the main topics such as automatic and closed-circuit systems, pharmacological agents used in low-flow anesthesia, drug management, effects on organ systems, anesthesia techniques and application methods. In addition, it has also helped to identify the main themes such as the application, monitoring, control and application methods of anesthetic gases.

Unlike conventional approaches such as meta-analyses and systematic reviews, bibliometric analyses focus on revealing research trends, keyword dynamics, and collaboration patterns rather than directly evaluating the outcomes of existing studies. In contrast, systematic reviews and meta-analyses are secondary research methods aimed at informing scientific knowledge production and practice; while systematic reviews provide a qualitative synthesis of existing studies, meta-analyses statistically combine the results of those studies to establish a quantitative level of evidence [28,29]. In this context, rather than directly assessing outcomes, bibliometric analyses reveal the intellectual structure and temporal evolution of the literature, offering data-driven insights into future research directions. In the present study, to identify emerging topics over time, the method of evaluating keywords according to their timeline described by Yao as “trend topics” was adopted [30]. The fact that the terms “closed loop” and “performance” are trending shows that automatic control systems and performance improvement studies in low-flow anesthesia remain up to date. The frequent use of pharmacological agents such as “propofol” and “remifentanil” reflects the interest in studies on drug management and efficacy; the prominence of monitoring terms such as “bispectral index” and “EEG analysis” shows a tendency to measure the depth of anesthesia and patient responses more precisely and indicates that anesthesia performance optimization as well as temperature control are becoming important. Among the trend keywords identified in our study, terms such as “propofol”, “remifentanil” and “closed-loop” are not specific to inhalation anesthesia but are rather associated with TIVA and automation systems, and should be interpreted as data-driven findings derived from the bibliometric analysis. This indicates that the low-flow anesthesia literature has evolved over time in interaction with different anesthesia techniques and technological approaches. In addition, the fact that the terms “FGF” (fresh gas flow) and “absorbent” attract attention is an indicator of the growing interest in low gas flow rates and environmentally friendly applications. For researchers working in this field, it is thought that it will be useful to focus on issues such as providing stable and safe anesthesia levels, optimizing anesthesia management, providing economic and environmental benefits, and improving patient safety. The emergence of terms such as “closed-loop” and “bispectral index” reflects the integration of automated control systems and advanced monitoring into clinical practice, supporting precision-guided and patient specific anesthesia management. This aligns with the growing global emphasis on sustainable healthcare practices and eco-conscious anesthetic choices.

Prior methodological work has emphasized that bibliometric findings can vary substantially depending on which WoSCC sub dataset is queried and comparative studies of WoS and Scopus have shown systematic differences in journal coverage and citation counts between the two databases [13,14]. The restriction of the data analyses in our study to the SCIE of WoSCC has resulted in the exclusion of other relevant publications indexed in other WoSCC sub-databases or alternative databases such as Scopus. The retrospective design of our study and the inclusion of publications only within a specific time frame may have limited the comprehensive evaluation of studies reflecting the most recent advances in low-flow anesthesia. The literature indicates that non-English publications have a relatively limited and changing role in scientific communication indexed within the SCIE of WoSCC [31]. Similarly, our inclusion of only English-language publications in the analysis may have led to the exclusion of relevant studies published in other languages. Previous studies indicate that older records in WoSCC may be affected by historical coverage discrepancies, retrospective indexing modifications, and missing metadata [15]. Therefore, the possibility of temporal inconsistencies should be considered when interpreting the findings of the current study. Previous research indicates that missing author affiliations in the SCIE of WoSCC can lead to biased institutional bibliometric indicators [32]. Accordingly, the possibility of a similar bias should be acknowledged in the context of our study. Additionally, variability in keyword usage (e.g., failure to capture synonyms), combined with the restriction of the search strategy to predefined keywords and specific indexing fields, may have introduced classification bias and caused some relevant studies to be overlooked. Moreover, institutional subscription and access restrictions may affect the accessibility of certain studies, thereby limiting the scope and reproducibility of the analysis. Although bibliometric analyses provide valuable insights into research productivity and structural trends, they do not directly assess the methodological quality of individual studies. Therefore, the findings should be interpreted within the inherent limitations of bibliometric methodology.

Despite these limitations, we believe that this study offers valuable insights for researchers working in the field of low-flow anesthesia. Especially in an area like low-flow anesthesia, which encompasses both clinical applications and environmental impact, identifying research trends can serve as a guide for the planning of future studies. These findings can support institutional policies aimed at reducing the carbon footprint of anesthetic practices by highlighting research priorities such as optimizing fresh gas flow, improving gas absorption systems, and adopting automated delivery technologies.

## 5. Conclusions

This bibliometric study provides a comprehensive overview of the scientific landscape surrounding low-flow anesthesia over the past three decades. The analysis identified key thematic trends concentrated around closed-loop systems, pharmacological agents such as remifentanil and sevoflurane, monitoring tools like bispectral index and EEG analysis, and environmental aspects including FGF and absorbent systems. These findings indicate that low-flow anesthesia is not only a cost effective and clinically efficient practice but also an evolving field aligned with global sustainability goals. Future research should prioritize the development of AI-assisted automated anesthesia delivery systems, the optimization of FGF to reduce waste and emissions, and the advancement of monitoring technologies to enhance patient safety. In addition, future studies should focus on integrating AI-based decision support systems into low-flow anesthesia practice, developing standardized protocols for safe and sustainable fresh gas flow management, and conducting high-quality clinical and translational studies to validate the clinical effectiveness of emerging technologies.

## Figures and Tables

**Table 1:** Search strategy for Web of Science database.

Component	Details
Database	SCIE of WoSCC
Search Date	15 September 2024
Time Frame	1993–2024
Language	English only
Document Types	Articles and review articles
Keyword Selection	Based on expert consensus among anesthesiologists with ≥10 years of experience
Search Fields	Title (TI) and Author Keywords (AKs)
Keywords Used 1	“AK = (Reduced flow)” OR “TI = (Reduced flow)” OR “TI = (Rebreathing system)” OR “AK = (Rebreathing system)” OR “AK = (Closed system)” OR “TI = (Closed system)” OR “TI = (Low flow)” OR “AK = (Low flow)” OR “AK = (Minimal flow)” OR “TI = (Minimal flow)”
Keywords Used 2	“TI = (Anesthesia)” OR “AK = (Anesthesia)” OR “TI = (Anesthetics)” OR “AK = (Anesthetics)” OR “TI = (Sevoflurane)” OR “AK = (Sevoflurane)” OR “TI = (Desflurane)” OR “AK = (Desflurane)” OR “TI = (Isoflurane)” OR “AK = (Isoflurane)”
Boolean Combination	Keywords Used 1 AND Keywords Used 2
Exclusion Criteria	Veterinary publications, duplicate records, incomplete bibliographic data, and non-English articles

Science Citation Index Expanded (SCIE); Web of Science Core Collection (WoSCC).

**Table 2:** Top 10 most productive contributors (country/institution/journal/author).

Rank	Country	NP	Institution	NP	Journal	NP	Author	NP
1	USA	39	Northwestern University	14	*Anesthesia & Analgesia*	24	André M. De Wolf	15
2	Turkey	33	Ghent University	13	*Anesthesiology*	23	Jan F. A. Hendrickx	15
3	Japan	27	Univ. of Washington	12	*Acta Anaesthesiologica Scandinavica*	19	Hiromichi Bito	11
4	Belgium	16	Univ. of Washington, Seattle	12	*Anaesthesia*	19	Rik Carette	11
5	Germany	16	Feinberg School of Medicine	11	*J. Clinical Monitoring & Computing*	16	Kazuyuki Ikeda	8
6	Sweden	14	Univ. of British Columbia	11	*Anaesthesia & Intensive Care*	15	Gordon G. Lockwood	7
7	UK	14	KU Leuven	8	*British Journal of Anaesthesia*	15	Hans H. Luttropp	7
8	Canada	10	Lund University	8	*Journal of Clinical Anesthesia*	12	Evan D. Kharasch	6
9	China	9	McGill University	8	*Appl. Cardiopulmonary Pathophysiology*	9	Sofie De Cooman	5
10	Australia	8	Univ. of Toronto	8	*European Journal of Anaesthesiology*	7	Thierry Deloof	5

NP: Number of publications.

**Table 3:** Most cited countries, institutions, journals, authors, and articles.

Rank	Country	NC	Institution	NC	Journal	NC	Author	NC
1	USA	929	Northwestern University	14	*Anesthesiology*	912	Hiromichi Bito	99
2	Japan	703	Ghent University	13	*Anesthesia & Analgesia*	779	Kazuyuki Ikeda	87
3	Germany	338	Univ. of Washington	12	*British Journal of Anaesthesia*	321	Evan D. Kharasch	51
4	Sweden	269	Univ. of Washington, Seattle	12	*Anaesthesia*	232	Eric J. Frink	43
5	UK	232	Feinberg School of Medicine	11	*Acta Anaesthesiologica Scandinavica*	274	André M. De Wolf	29
6	Turkey	206	Univ. of British Columbia	11	*Anaesthesia & Intensive Care*	154	Jan F. A. Hendrickx	29
7	Italy	163	KU Leuven	8	*European Journal of Anaesthesiology*	139	Alan A. Artru	26
8	Belgium	150	Lund University	8	*J. Clinical Monitoring & Computing*	123	Yukako Ikeuchi	26
9	Canada	136	McGill University	8	*Anaesthesist*	141	Thomas J. Ebert	24
10	France	122	Univ. of Toronto	8	*Lancet*	75	Rik Carette	22

NC: Number of citations.

**Table 4:** Centrality metrics of influential publications.

Node	PageRank	Betweenness	Closeness	Cluster	Color
Hiromichi Bito 1994: Long-duration, low-flow sevoflurane anesthesia using two carbon dioxide absorbents. Quantification of degradation products in the circuit.	0.033	47.994	0.003	3	Green
Evan D. Kharasch 1997: Assessment of low-flow sevoflurane and isoflurane effects on renal function using sensitive markers of tubular toxicity.	0.032	0.687	0.002	3	Green
Michio Morio 1992: Reaction of sevoflurane and its degradation products with soda lime. Toxicity of the byproducts.	0.03	17.341	0.003	3	Green
Christopher T. Gonsowski 1994: Toxicity of compound A in rats. Effect of a 3 h administration.	0.03	17.622	0.003	3	Green
Eric J. Frink 1992: Clinical comparison of sevoflurane and isoflurane in healthy patients.	0.028	354.139	0.003	3	Green
Hiromichi Bito 1995: Degradation products of sevoflurane during low-flow anesthesia.	0.027	56.102	0.003	3	Green
Alan D. Baxter 1997: Low- and minimal flow inhalational anesthesia.	0.027	260.571	0.003	1	Red
Jan A. Baum 1995: Low-flow anesthesia.	0.027	56.953	0.003	1	Red
Richard B. Weiskopf 1993: Comparing the costs of inhaled anesthetics.	0.025	14.813	0.002	1	Red
Hiromichi Bito 1997: Effects of low-flow sevoflurane anesthesia on renal function: comparison with high-flow sevoflurane anesthesia and low-flow isoflurane anesthesia.	0.025	0.404	0.002	3	Green
Murat Bilgi 2011: Comparison of the effects of low-flow and high-flow inhalational anesthesia with nitrous oxide and desflurane on mucociliary activity and pulmonary function tests.	0.025	67.856	0.003	1	Red
Edmund I. Eger II 1997: Baralyme dehydration increases and soda lime dehydration decreases the concentration of compound A resulting from sevoflurane degradation in a standard anesthetic circuit.	0.024	0.208	0.002	3	Green
Michel M. R. F. Struys 2001: Target-controlled administration of inhaled anesthetics.	0.024	0.147	0.002	2	Blue
Ngai Liu 2011: Closed-loop coadministration of propofol and remifentanil guided by bispectral index: a randomized multicenter study.	0.022	0.22	0.002	2	Blue
Ngai Liu 2012: Review article: closed-loop systems in anesthesia: is there a potential for closed-loop fluid management and hemodynamic optimization?	0.022	0.183	0.002	2	Blue

**Table 5:** Centrality metrics of influential authors.

Node	PageRank	Betweenness	Closeness	Cluster	Color
Edmund I. Eger II	0.046	580.967	0.01	2	Blue
Hiromichi Bito	0.039	20.685	0.007	3	Green
Jan A. Baum	0.033	32.833	0.008	2	Blue
Eric J. Frink	0.033	103.913	0.008	3	Green
Ngai Liu	0.031	10.228	0.007	1	Red
Evan D. Kharasch	0.031	51.26	0.007	3	Green
William W. Mapleson	0.03	16.548	0.008	2	Blue
Jan Baum	0.03	66.573	0.008	2	Blue
Hideyuki Higuchi	0.028	11.34	0.008	3	Green
Michio Morio	0.028	2.669	0.007	3	Green
Michel M. R. F. Struys	0.027	109.803	0.008	1	Red
Jan F. A. Hendrickx	0.027	62.865	0.008	2	Blue
Robert I. Mazze	0.027	41.535	0.008	3	Green
Girish D. Puri	0.026	7.198	0.007	1	Red
Jan P. Bengtson	0.026	34.251	0.007	2	Blue

**Table 6:** Centrality criteria of influential journals.

Node	PageRank	Betweenness	Closeness	Cluster	Color
*Anesthesiology*	0.099	82.163	0.013	2	Blue
*Anesthesia & Analgesia*	0.093	76.151	0.013	2	Blue
*British Journal of Anaesthesia*	0.088	56.411	0.013	2	Blue
*Anaesthesia*	0.071	32.862	0.012	2	Blue
*Acta Anaesthesiologica Scandinavica*	0.058	13.374	0.012	2	Blue
*Journal of Clinical Anesthesia*	0.036	6.328	0.012	2	Blue
*European Journal of Anaesthesiology*	0.035	9.204	0.011	2	Blue
*Canadian Journal of Anesthesia*	0.034	7.905	0.012	2	Blue
*Anaesthesia and Intensive Care*	0.031	488.373	0.016	3	Green
*Anaesthesist*	0.023	2.137	0.011	2	Blue
*Journal of Clinical Monitoring & Computing*	0.021	118.997	0.013	3	Green
*Lancet*	0.02	0.775	0.011	2	Blue
*New England Journal of Medicine*	0.02	5.924	0.012	2	Blue
*Minerva Anestesiol*	0.016	1.322	0.011	2	Blue
*Journal of Applied Physiology*	0.015	0.885	0.011	2	Blue

**Table 7:** Thematic clusters identified in the MDS analysis of author keywords, Keyword Plus terms, and abstract words.

Figures	Groups	Keywords
MDS Analysis of Author Keywords (Figure 7)	Blue	automated anesthesia, low flow, desflurane, closed circuit
	Red	closed loop, anesthesia machine, target control, automated low-flow, equipment
	Green	nitrous oxide, vaporizer, circle system, nitrogen, oxygen
MDS Analysis of Keyword Plus Words (Figure 8)	Blue	inhaled anesthetics, heat, absorption, metabolism, compound, humidity, degradation
	Red	breathing systems, circuit, baralyme, hepatic function, rats, isoflurane anesthesia, renal function
	Green	induction, bispectral index, delivery system, feedback control, quantitative EEG analysis, titration, feasibility
MDS Analysis of Abstract Words (Figure 9)	Blue	alveolar concentration, flow rate, canister life, carbon dioxide, soda lime, breathing circuit, degradation products, vaporizer setting
	Red	low-flow isoflurane, serum creatinine, isoflurane anesthesia, high-flow sevoflurane, renal function, significant differences, minimum alveolar, low-flow sevoflurane
	Green	intravenous anesthesia, arterial pressure, heart rate, patients undergoing, significantly lower, control system, bispectral index, laparoscopic cholecystectomy

## Data Availability

The data presented in this study were obtained from the SCIE of WoSCC, a publicly available database.

## Abbreviations

The following abbreviations are used in this manuscript:

AKs	Author Keywords
BIS	Bispectral Index
CO_2_	Carbon Dioxide
DOI	Digital Object Identifier
EEG	Electroencephalography
FGF	Fresh Gas Flow
MDS	Multidimensional Scaling
N_2_O	Nitrous Oxide
SCIE	Science Citation Index Expanded
TI	Title
TIVA	Total Intravenous Anesthesia
USA	United States of America
UK	United Kingdom
WoS	Web of Science
WoSCC	Web of Science Core Collection
